# Hospital-level variation in follow-up strategies after percutaneous coronary intervention, revealed in health claims data of Korea

**DOI:** 10.1038/s41598-021-82960-4

**Published:** 2021-02-08

**Authors:** Jae-Hyung Roh, Jihyun Sohn, Jae-Hwan Lee, In-Sun Kwon, Hanbyul Lee, Yong-Hoon Yoon, Minsu Kim, Yong-Giun Kim, Gyung-Min Park, Jong-Young Lee, Jae-Hyeong Park, Dong Heon Yang, Hun Sik Park

**Affiliations:** 1 Department of Cardiology in Internal Medicine, School of Medicine, Cardiovascular Center, Chungnam National University, Chungnam National University Hospital, 282 Munhwa-ro, Jung-gu, Daejeon, Korea; 2 Department of Internal Medicine, Kyungpook National University School of Medicine, Cardiology Center, Kyungpook National University Chilgok Hospital, Daegu, Korea; 3 Clinical Trials Center, Chungnam National University Hospital, Daejeon, Korea; 4 Department of Statistics, Kyungpook National University, Daegu, Korea; 5 Department of Cardiology, University of Ulsan College of Medicine, Ulsan University Hospital, Ulsan, Korea; 6 Division of Cardiology, Department of Internal Medicine, Kangbuk Samsung Hospital, Sungkyunkwan University School of Medicine, Seoul, Korea; 7 Division of Cardiology, Kyungpook National University Hospital, Daegu, Korea

**Keywords:** Cardiology, Interventional cardiology

## Abstract

This study sought to determine hospital variation in the use of follow-up stress testing (FUST) and invasive coronary angiography (FUCAG) after percutaneous coronary intervention (PCI). The claims records of 150,580 Korean patients who received PCI in 128 hospitals between 2008 and 2015 were analyzed. Patient were considered to have undergone FUST and FUCAG, when these testings were performed within two years after discharge from the index hospitalization. Hierarchical generalized linear and frailty models were used to evaluate binary and time-to-event outcomes. Hospital-level risk-standardized FUCAG and FUST rates were highly variable across the hospitals (median, 0.41; interquartile range [IQR], 0.27–0.59; median, 0.22; IQR, 0.08–0.39, respectively). The performances of various models predicting the likelihood of FUCAG and FUST were compared, and the best performance was observed with the models adjusted for patient case mix and individual hospital effects as random effects (receiver operating characteristic curves, 0.72 for FUCAG; 0.82 for FUST). The intraclass correlation coefficients of the models (0.41 and 0.68, respectively) indicated that a considerable proportion of the observed variation was related to individual institutional effects. Higher hospital-level FUCAG and FUST rates were not preventive of death or myocardial infarction. Increased repeat revascularizations were observed in hospitals with higher FUCAG rates.

## Introduction

Percutaneous coronary intervention (PCI) has become one of the most frequently performed therapeutic procedures in medicine^1^. Now that many studies have shown that drug-eluting stents (DESs) were more effective in the prevention of restenosis than bare-metal stents (BMSs)^1^, the need to detect restenosis after PCI has reduced. Nevertheless, in daily practice, concerns still remain regarding the potential recurrence of symptoms or ischemia caused by disease progression or restenosis, especially after PCIs involving high risk lesion subsets (e.g. unprotected left main disease, bifurcation lesion, or chronic total occlusion) and those performed on patients with restenosis-prone conditions (e.g. diabetes mellitus). Unfortunately, recent clinical guidelines do not provide consistent recommendations for the optimal follow-up strategy after PCI^2,3^. Controversy surrounding this issue might lead to substantial practice-pattern variations and the excessive use of follow-up stress testing (FUST) and follow-up invasive coronary angiography (FUCAG). The extent of hospital variation in the use of these testing methods, however, remains largely unknown. Quantifying hospital variation in post-PCI follow-up strategies and its association with hospital factors and clinical outcomes is important to understand whether the indiscriminate application of these modalities is pervasive across hospitals or isolated to specific hospital groups. To address this issue, we used claims data of the National Health Insurance (NHI) in South Korea to examine hospital-level variations in the use of FUST and FUCAG after PCI, and whether certain hospital characteristics were associated with higher or lower rates of use. Moreover, we examined the implications of hospital variations by determining the association between a hospital’s rate of follow-up testing and clinical outcomes, such as mortality, myocardial infarction (MI), and repeat revascularization. 

## Results

### Patient characteristics

**Figure 1:**
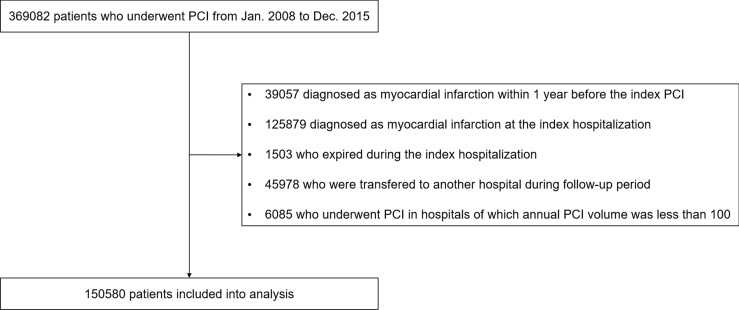
Overview of the study population. *PCI* percutaneous coronary intervention.

A total of 369,082 patients who underwent PCI between January 2008 and December 2015 were identified from the HIRA claims database. The exclusion of 218,502 patients who did not meet the eligibility criteria left 150,580 patients in 128 hospitals in South Korea in the study analysis (Fig. 1). Of these hospitals, 82% had more than 500 beds, 72.7% were teaching hospitals, and 53.9% were located in urban settings. The median annual volume of PCIs per hospital was 339.7 (IQR, 188.9–521.5). The baseline clinical characteristics of the enrolled patients are shown in Table 1. The hospitals were categorized into quartiles by per-hospital FUST and FUCAG rates [low (first quartile), low-to-medium (second quartile), medium-to-high (third quartile), and high (fourth quartile)].

**Table 1:** Baseline characteristics of the study patients.

Variables	Overall N = 150,580
Age, years	64.72 ± 10.6
Male	97,063 (64.5)
Diabetes	44,405 (29.5)
Diabetes with chronic complications	29,554 (19.6)
Hyperlipidemia	73,355 (48.7)
Hypertension	106,296 (70.6)
Congestive heart failure	12,511 (8.3)
Arrhythmia	13,576 (9.0)
Valvular heart disease	2493 (1.7)
Peripheral vascular disease	16,579 (11.0)
Cerebrovascular disease	18,477 (12.3)
Chronic pulmonary disease	22,197 (14.7)
Chronic kidney disease	7316 (4.9)
Charlson comorbidity index	2.0 ± 1.9

Values are presented as number (percentage) or mean ± standard deviation.

### Hospital-level variation in follow-up strategy after PCI

**Figure 2:**
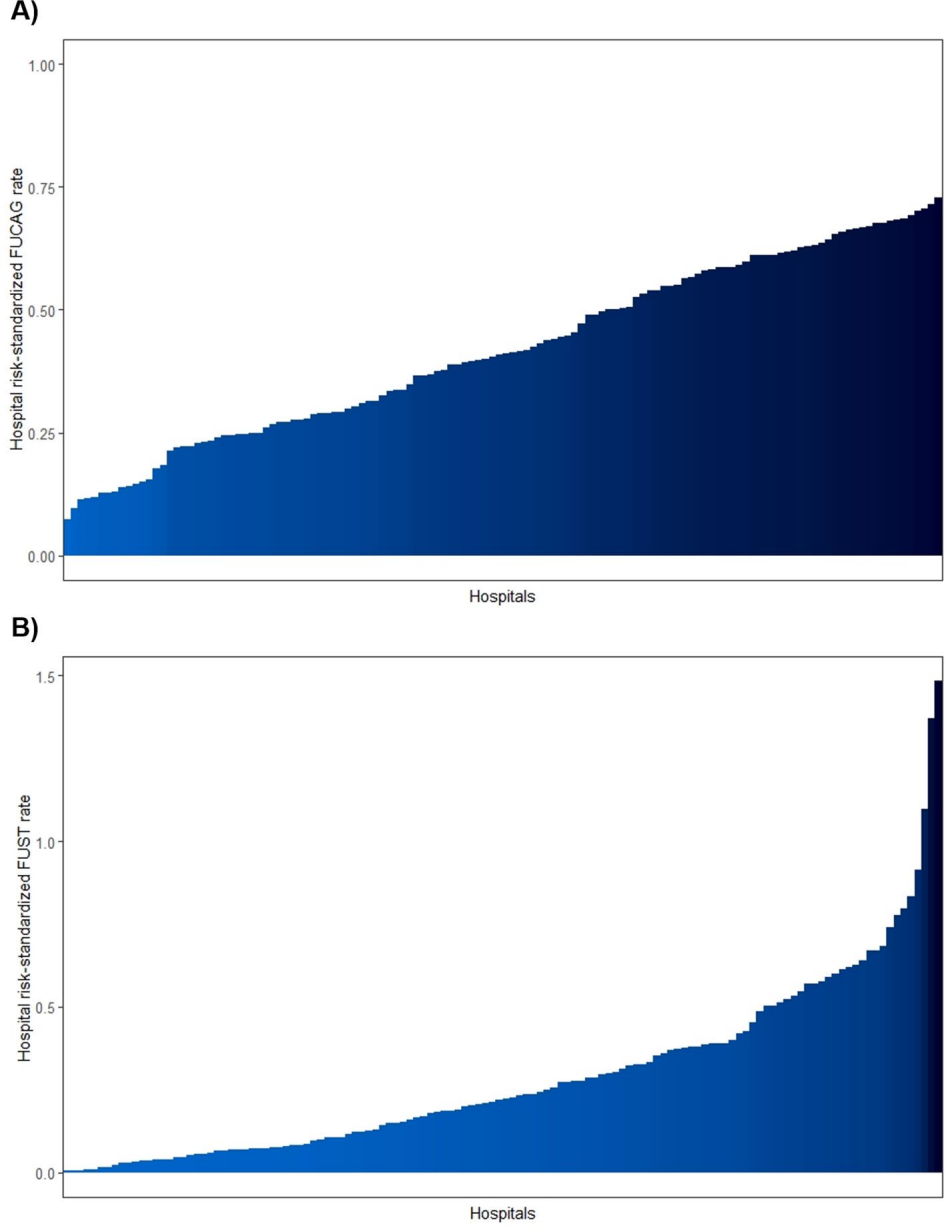
Risk-standardized rates of follow-up strategies after percutaneous coronary intervention across hospitals. (**A**) Graph showing hospital-level risk-standardized rates of FUCAG. (**B**) Graph showing hospital-level risk-standardized rates of FUST. *FUCAG* follow-up invasive coronary angiography, *FUST* follow-up stress testing. The figure was created with R (version 3.5.1)^4^.

**Figure 3:**
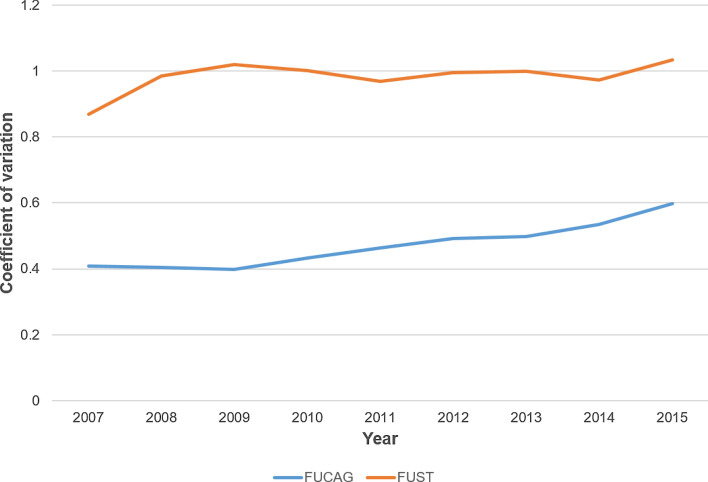
Hospital-level variations in follow-up strategy over years. *FUCAG* follow-up invasive coronary angiography, *FUST* follow-up stress testing.

The unadjusted median FUCAG and FUST rates were 34.0% (IQR, 22.5–44.9%) and 10.8% (IQR, 4.3–19.2%), respectively. The median time-gaps from the index hospitalization to FUCAGs and FUST was 324 days (IQR, 245–385 days), and 318 days (IQR, 157–422 days), respectively. As shown in Fig. 2, the hospital-level risk-standardized FUCAG and FUST rates were highly variable across the hospitals (median, 0.41; IQR, 0.27–0.59 for FUCAG; median, 0.22; IQR, 0.08–0.39 for FUST). Time-trends of the between-hospital variation in FUCAG and FUST rates are depicted in Fig. 3. Unlike FUST, where the between-hospital variation maintained stable, that of FUCAG increased over time. Table 2 depicts the hospital characteristics according to follow-up strategies after PCI. Hospitals with the low FUCAG rates were more likely to be teaching hospitals and to perform FUST than those with the high FUCAG rates. The risk-standardized death rates were significantly different across the FUCAG quartiles, but a tendency was not discernible across the FUCAG quartile groups. Hospitals with high FUCAG rates demonstrated higher risk-standardized rates of composite outcomes, which were mainly derived from the increased risk-standardized repeat revascularization rates of these hospitals. FUST was more frequently performed in hospitals having a higher number of beds, or providing medical education. The risk-standardized rates of the composite outcomes were significantly lower in hospitals with high FUST rates, but no significant difference was observed for each element of the composite outcome across the FUST quartiles.

**Table 2:** Hospital characteristics according to follow-up strategy after percutaneous coronary intervention.

	Low (1st quartile)	Low-to-medium (2nd quartile)	Medium-to-high (3rd quartile)	High (4th quartile)	*p* value
**FUCAG rate**					
No. of hospitals*	32 (25.0)	32 (25.0)	32 (25.0)	32 (25.0)	
Teaching hospitals*	29 (31.2)	24 (25.8)	24 (25.8)	16 (17.2)	0.003
Urban*	21 (30.4)	17 (24.6)	17 (24.6)	14 (20.3)	0.37
Risk-standardized FUST rate^†^	0.495 (0.244–0.656)	0.159 (0.067–0.324)	0.231 (0.076–0.324)	0.114 (0.056–0.235)	< 0.001
Beds (No.) ^†^	840.0 (706.0–1050.0)	739.0 (590.0–981.5)	670.0 (542.5–871.0)	676.0 (432.0–949.8)	0.089
Annual PCI volume^†^	394.0 (198.3–538.1)	336.2 (180.0–544.1)	305.6 (195.9–495.5)	312.3 (188.1–554.0)	0.84
RSDR^†^	0.078 (0.064–0.082)	0.067 (0.062–0.076)	0.078 (0.069–0.085)	0.069 (0.061–0.079)	0.039
RSMR^†^	0.056 (0.046–0.064)	0.051 (0.044–0.059)	0.054 (0.047–0.068)	0.049 (0.041–0.054)	0.16
RSRR^†^	0.125 (0.117—0.157)	0.144 (0.126–0.162)	0.160 (0.144–0.206)	0.167 (0.154–0.202)	< 0.001
RSCR^†^	0.212 (0.199–0.239)	0.219 (0.209–0.239)	0.249 (0.221–0.289)	0.248 (0.227–0.269)	< 0.001
**FUST rate**					
No. of hospitals*	32 (25.0)	32 (25.0)	32 (25.0)	32 (25.0)	
Teaching hospitals*	15 (27.1)	26 (28.8)	23 (25.4)	29 (18.6)	< 0.001
Urban*	16 (23.2)	15 (21.7)	17 (24.6)	21 (30.4)	0.46
Risk-standardized FUCAG rate^†^	0.481 (0.356–0.634)	0.511 (0.314–0.641)	0.408 (0.283–0.522)	0.256 (0.165–0.459)	< 0.001
Beds (No.) ^†^	557.3 (427.0–739.5)	797.5 (542.5–985.5)	714.3 (615.5–1006.0)	889.0 (695.5–1050.0)	< 0.001
Annual PCI volume^†^	226.8 (161.4–416.9)	416.0 (213.2–526.7)	339.7 (200.8–464.4)	413.1 (243.9–667.8)	0.077
RSDR^†^	0.072 (0.065–0.079)	0.073 (0.066–0.083)	0.071 (0.063–0.084)	0.072 (0.059–0.080)	0.83
RSMR^†^	0.051 (0.047–0.056)	0.052 (0.049–0.058)	0.059 (0.048–0.067)	0.050 (0.042–0.062)	0.26
RSRR^†^	0.152 (0.144–0.198)	0.159 (0.140–0.183)	0.153 (0.125–0.177)	0.142 (0.122–0.168)	0.061
RSCR^†^	0.235 (0.214–0.268)	0.244 (0.215–0.269)	0.232 (0.210–0.267)	0.216 (0.204–0.244)	0.047

*Data are expressed as number (percentage).

^†^Data are expressed as median (interquartile range).

*FUCAG* follow-up invasive coronary angiography, *FUST* follow-up stress testing, *PCI* percutaneous coronary intervention, *RSCR* risk-standardized the composite outcome (a composite of all-cause death, myocardial infarction, or repeat revascularization) rate, *RSDR* risk-standardized death rate, *RSMR* risk-standardized myocardial infarction rate, *RSRR* risk-standardized repeat revascularization rate.

### Hospital factors associated with follow-up strategies after PCI

After adjusting for patient characteristics, we assessed the association between follow-up pattern and hospital characteristics by adding the following hospital characteristics to the HGLM models: annual PCI volume, size, urban versus rural setting, and teaching versus nonteaching status (Table 3). FUCAGs were performed more frequently in hospitals with a lower number of beds, those located in rural areas, and those not providing medical education. However, the odds of performing FUST were higher in hospitals with a higher annular PCI volume, a higher number of beds, and in teaching hospitals. The addition of hospital characteristics to the logistic regression models was associated with improvement in the performance of the models for FUCAGs and FUST (Table 4). However, the best performance was obtained with HGLM models adjusted for patient factors and individual hospital effects as random effects. Furthermore, the intraclass correlation coefficients (ICC) of the HGLM indicated that a noteworthy proportion of the variation in follow-up patterns could be explained by individual institutional effects after accounting for differences in patient characteristics. Individual institutional effects could explain 41% of the variability in hospital-level FUCAG rates, and 68% of the variability in hospital-level FUST rates.

**Table 3:** Hospital factors associated with follow-up strategy after percutaneous coronary intervention.

Variables	FUCAG	FUST
	OR (95% CI)	OR (95% CI)
**Annual PCI volume**		
Low (1st quartile)	1.14 (0.76–1.73)	0.45 (0.23–0.90)
Low-to-medium (2nd quartile)	1.13 (0.75–1.71)	0.49 (0.25–0.96)
Medium-to-high (3rd quartile)	0.82 (0.54–1.24)	1.39 (0.71–2.73)
High (4th quartile)	Reference	
**No. of beds**		
Low (1st quartile)	1.68 (1.13–2.51)	0.22 (0.11–0.43)
Low-to-medium (2nd quartile)	1.60 (1.07–2.39)	0.45 (0.24–0.88)
Medium-to-high (3rd quartile)	0.99 (0.66–1.47)	0.92 (0.48–1.77)
High (4th quartile)	Reference	
Urban (vs. rural)	0.73 (0.55–0.98)	1.33 (0.80–2.20)
Teaching hospital (vs. nonteaching)	0.55 (0.40–0.75)	3.49 (2.05–5.94)

*CI* confidence interval, *FUCAG* follow-up invasive coronary angiography, *FUST* follow-up stress testing, *OR* odds ratio, *PCI* percutaneous coronary intervention.

**Table 4:** Importance of institutional factors in the variation of follow-up strategies.

	No. of patients, N (%)	ROC curve without insitutional effect*	ROC curve with general institutional characteristics^†^	ROC curve with institutional effect^‡^	ICC
FUCAG	60,226 (40.0%)	0.577	0.617	0.721	0.410
FUST	28,567 (19.0%)	0.631	0.679	0.816	0.680

*Logistic regression models including patient characteristics as covariates.

^†^Logistic regression models including patient characteristics and hospital characteristics as covariates.

^‡^Hierarchical logistic regression models including patient characteristics and hospital random effects.

*FUCAG* follow-up invasive coronary angiography, *FUST* follow-up stress testing, *ICC* intraclass correlation coefficient, *ROC* receiver operating characteristic.

### Hospital factors associated with clinical outcomes

During the median 56 months of follow-up, 35,515 (23.6%) patients experienced the primary outcome of interest [all-cause death, 10,917 (7.2%) patients; MI, 7958 (5.3%) patients; repeat revascularization, 23,249 (15.4%) patients]. The association of hospital characteristics and follow-up patterns with clinical outcomes was evaluated by adding each of these hospital-level variables into the models, which were adjusted for patient characteristics (Table 5). The risk for the composite outcome was observed to be lower in hospitals with low FUCAG rates, which mainly resulting from the lower risk for repeat revascularization in these hospitals. As for death and MI, despite statistically significant HRs in some quartiles, there was no remarkable trend of HRs across the FUCAG quartiles. No significant association was observed between hospital-level FUST rates and the risk for hard endpoints, such as all-cause death, and MI. Unlike FUCAGs, there was a negative relationship between FUST and repeat revascularization rates, resulting in higher rates of the composite outcome in hospitals with low FUST rates.

**Table 5:** Hospital factors associated with clinical outcomes.

Variables	Death	Myocardial infarction	Repeat revascularization	Composite outcome
	HR (95% CI)	HR (95% CI)	HR (95% CI)	HR (95% CI)
Annual PCI volume*				
Low	1.19 (1.05–1.35)	1.18 (1.01–1.39)	1.14 (0.98–1.33)	1.17 (1.05–1.30)
Low-to-medium	1.27 (1.13–1.42)	1.17 (1.01–1.36)	1.01 (0.87–1.18)	1.10 (0.99–1.22)
Medium-to-high	1.09 (0.98–1.22)	1.15 (0.99–1.33)	0.95 (0.82–1.10)	1.01 (0.91–1.12)
High	Reference			
No. of beds*				
Low	1.08 (0.95–1.23)	1.09 (0.93–1.28)	1.28 (1.11–1.47)	1.22 (1.10–1.35)
Low-to-medium	1.08 (0.95–1.23)	1.13 (0.97–1.32)	1.12 (0.98–1.30)	1.13 (1.02–1.25)
Medium-to-high	1.04 (0.92–1.18)	0.99 (0.85–1.15)	0.97 (0.84–1.12)	1.01 (0.92–1.12)
High	Reference			
Urban (vs. rural)	1.03 (0.94–1.13)	1.03 (0.92–1.15)	1.03 (0.92–1.15)	1.01 (0.94–1.10)
Teaching (vs. nonteaching)	1.09 (0.98–1.20)	1.04 (0.92–1.19)	0.82 (0.73–0.92)	0.90 (0.83–0.98)
FUCAG*				
Low	1.10 (0.98–1.25)	1.19 (1.02–1.39)	0.70 (0.61–0.80)	0.82 (0.75–0.91)
Low-to-medium	0.96 (0.85–1.09)	1.05 (0.90–1.23)	0.76 (0.66–0.87)	0.83 (0.75–0.92)
Medium-to-high	1.15 (1.02–1.30)	1.14 (0.98–1.33)	0.93 (0.82–1.07)	0.99 (0.90–1.10)
High	Reference			
FUST*				
Low	1.02 (0.90–1.16)	1.03 (0.88–1.21)	1.26 (1.09–1.46)	1.17 (1.06–1.30)
Low-to-medium	1.10 (0.97–1.25)	1.02 (0.87–1.19)	1.22 (1.05–1.41)	1.17 (1.06–1.30)
Medium-to-high	1.07 (0.94–1.21)	1.16 (0.99–1.35)	1.12 (0.97–1.30)	1.12 (1.01–1.24)
High	Reference			

*Low indicates first quartile, low-to-medium second quartile, medium-to-high third quartile, and high fourth quartile.

*CI* confidence interval, *FUCAG* follow-up invasive coronary angiography, *FUST* follow-up stress testing, *HR* hazard ratio, *PCI* percutaneous coronary intervention.

## Discussion

In this large retrospective cohort study, we investigated hospital-level variations in follow-up strategies after PCI and their association with clinical outcomes. Marked differences were observed in hospital-level FUCAG and FUST rates among a diverse group of hospitals in South Korea. Variations in hospital-level FUCAG and FUST use reflected the differences in the hospitals as well as patient characteristics. No remarkable association was observed between hospital-level FUCAG and FUST use and the occurrence of hard endpoints, such as all-cause death, and MI. However, patients who received PCI in hospitals with high FUCAG rates were more likely to experience repeat revascularization than those in hospitals with lower rates.

After PCI, 18% of the patients who received PCIs were reported to experience angina symptoms at the 1-year follow-up in a study analyzing data from the National Heart, Lung, and Blood Institute’s Dynamic registry^5^. Given that the percentage of patients with recurrent angina is expected to have dropped considerably with the increased use of drug-eluting stents and the greater experience of operators in the interventional laboratory, our 34% hospital-level median FUCAG rate seems to be notably larger than would be predicted from the symptoms alone. Apart from the relatively high FUCAG rates, the hospital-level variability deserves more attention. Although FUCAG is advocated for patients with high-risk anatomical features (e.g. unprotected left main stenosis) in some guidelines^2^, a trend toward increasing variability over the years indicates that the high hospital-level variability in FUCAG rates was not solely attributable to the clinical features of patients who were admitted to the respective hospitals. In the current study, the performance of a statistical model to predict the likelihood a patient received a FUCAG was observed to improve when general institutional characteristics were added to the clinical features of patients as covariates. However, more emphasis should be placed on the fact that the model performed best when individual hospital effect were added as random effects. The high ICC indicated that the odds of a patient undergoing FUCAG markedly relied on unmeasured variables indigenous to the respective hospitals, apart from the clinical features of patients and general hospital characteristics. Similar results were observed with FUST, except that the likelihood of performing FUST was higher in hospitals with features opposite those favoring FUCAGs. It is reasonable to assume that unmeasured variables included physician attitudes and training, patient preferences, inappropriate use, and other nonclinical factors not captured in an administrative dataset. Considering that variability derived from these nonclinical factors potentially leads to the overuse of FUCAGs and FUST, they need to be adequately assessed and standardized by further research, education, and regulations. 

On the patient-level, several studies have reported a significant association between routine FUCAGs and an increased risk for repeat revascularization after PCI^6,7^. Recently, Misumida et al. performed a meta-analysis of five studies to compare the clinical outcomes after PCI between patients who underwent routine FUCAGs and those who only had clinical follow-ups and reported that routine FUCAGs were significantly associated with higher rates of repeat revascularization^8^. However, several factors potentially limited its reliability and generalizability. First, most of the studies included in the meta-analyses were not randomized specifically to reveal the role of FUCAGs. Second, a part of the studies was not performed in the DES era. Finally, the study population of the randomized clinical trials are not representative of real-world practices. To overcome these limitations, we analyzed a nationwide claims database which included practically all the PCI patients in South Korea contemporaneously, and found that a higher hospital-level FUCAG rate was significantly associated with a higher risk of repeat revascularization. Our findings on FUCAG were consistent with the concept of ischemia-guided revascularization suggested by the Fractional Flow Reserve Versus Angiography for Multivessel Evaluation trial, where fractional flow reserve-guided PCI was superior to angiography-guided PCI in terms of a composite endpoint of death, nonfatal MIs, and repeat revascularizations at 1-year^9^. As in the case of de novo coronary stenosis, it seems reasonable to assume that early detection of hemodynamically insignificant in-stent restenosis did not improve clinical outcomes, only increased repeat revascularizations. Similarly, no significant association between hospital-level FUST rates and the risks for death and MI was observed. Previous studies which found that the detection of silent ischemia on functional testing after PCI had no impact on clinical outcomes support these findings^10,11^. The increased risk for repeat revascularizations in hospitals with lower FUST rates was partly attributed to the higher FUCAG rates in these hospitals. 

Several limitations of the study need to be acknowledged. First, claims data from HIRA does not include clinical data such as angiographic and procedural characteristics, left ventricular ejection fraction, and laboratory and stress test results that are important determinants of follow-up strategies. However, despite the lack of these clinical and biologic data, the performance of the models showed that FUCAG and FUST use at the hospital level can be modeled adequately when accounting for both patient case mix and institutional clustering effects. Furthermore, given the findings that patient and hospital characteristics could only explain a small portion of hospital-level variability in FUCAG and FUST, performance of FUCAG and FUST was more likely affected by non-clinical factors rather than baseline information on coronary lesion, procedure characteristics, and stress tests. Second, the observed association between FUCAGs and increased repeat revascularizations does not necessarily indicate a causal relationship. Further studies will be needed to investigate this issue. Third, we used administrative data, which relies on ICD-10 codes, to identify patients and this data may suffer from misclassification biases. However, PCIs and coronary angiographies receive high levels of reimbursement and are unlikely to be misclassified. Fourth, coronary angiographies associated with staged PCI were not considered, but their effect on our results was deemed insignificant. The exclusion of MI patients at the index hospitalization ruled out the possibility of staged PCI for non-culprit lesions. The median time-gap between the index hospitalization and FUCAGs indicated that most FUCAGs were performed later than staged PCIs would be expected to be performed. Fifth, we could not identify early testing carried out for appropriate indications, such as FUCAGs for suspected MIs or FUST for new onset angina. As a result, our estimate of FUCAG and FUST incidence is conservative. Finally, our findings are based on data from South Korea, perhaps limiting the generalizability of our results to other ethnic groups and other reimbursement environments.

In conclusion, considerable variabilities in the rates of FUCAG and FUST after PCIs were observed across diverse hospitals in South Korea. The likelihood of a patient undergoing FUCAG or FUST was markedly dependent on which hospital the patient was admitted to. Higher hospital-level FUCAG and FUST rates did not seem to be preventive of the hard endpoints. However, an increased risk for repeat revascularization was observed in hospitals with higher FUCAG rates. 

## Methods

### Data sources

Under the control of the NHI system in South Korea, all health care providers submit claims and are reimbursed for medical services they provide on a fee-for service basis. The Health Insurance Review & Assessment Service (HIRA) is a quasigovernmental organization that systematically reviews all claims from health care providers to minimize the risk of redundant and unnecessary medical services. We conducted a retrospective cohort study using the claim records of the HIRA. Diagnosis codes from the International Classification of Diseases, 10th Revision (ICD-10) were used^12^. This study was approved by the local Institutional Review Board of the Chungnam National University Hospital, Daejeon, Korea, which waived the requirement for informed consent. All methods were performed in accordance with the guidelines and regulations of the ethic committee of Chungnam National University Hospital.

### Study population

From the claims database of the HIRA, we identified patients who underwent PCI from January 2008 to December 2015. This preliminary cohort represented approximately 100% of all PCI patients nationwide during the respective period. Subjects who met one of the following criteria were excluded: (1) patients who were diagnosed with MI (I21.X-I22.X) within one year before the index PCI, (2) patients whose primary diagnosis were MI at the index hospitalization, (3) patients who expired during the index hospitalization, (4) patients who were transferred to another hospital during the follow-up period, and (5) patients who underwent PCI in hospitals conducting less than 100 PCIs annually. The ICD-10 codes were also used to identify comorbid conditions^12,13^. The Charlson comorbidity index was obtained from the ICD-10 codes^12^.

### Follow-up strategies and clinical outcomes

To evaluate the follow-up strategies and clinical outcomes, we reviewed all claim data of the enrolled patients from January 2008 to December 2016. FUST consisted of exercise electrocardiography and myocardial perfusion scintigraphy. A patient was considered to have undergone FUST or FUCAG when these tests were performed within 2 years after discharge from the index hospitalization. The primary outcome of interest was a composite of all-cause death, MI, or repeat revascularization. We also examined each component of the primary outcome of interest. Death was identified from claims where the final state of a patient was coded as dead. MIs were identified from in-patient claims with MI in the diagnosis list. Repeat revascularization was defined as any PCI or coronary artery bypass grafting performed after discharge from the index hospitalization. The occurrence of FUST, FUCAG, PCI, and coronary artery bypass grafting was identified by HIRA-specific procedure codes.

### Statistical analysis

The results of descriptive analyses are presented as the mean ± standard deviation or the median (interquartile range [IQR]) for continuous variables, and number (percentage) for categorical data. The annual annual hospital-level variation in FUST and FUCAGs was evaluated using coefficients of variation. Continuous variables were compared with analysis of variance and categorical variables were compared with chi-square statistics or Fisher’s exact test, as appropriate.

We used hierarchical generalized linear models (HGLMs) to calculate the risk-standardized hospital rates of FUST, FUCAGs, and clinical outcomes^14,15^. We selected patient characteristics used as covariates for risk adjustment with a stepwise algorithm using logistic regression models. These rates were obtained as the ratio of the predicted to the expected hospital number of events, multiplied by the national unadjusted rate. The expected hospital events for each hospital is the number of given events expected in the hospital if the hospital’s patients were treated at a “reference” hospital. Operationally, it was calculated by regressing the risk factors on the respective events using all hospitals in our sample, applying the subsequently estimated regression coefficients to the patient characteristics observed in the hospital, then summing. The predicted hospital events is the number of expected events in the “specific” hospital and not at a reference hospital. Operationally, it was calculated by estimating a hospital-specific random effect that represented the baseline risk for the respective events within the hospital, applying the hospital-specific regression coefficients to the patient characteristics in the hospital, then summing. 

The association of hospital characteristics with hospital-level FUST and FUCAG use was evaluated using HGLMs. The models were adjusted for patient characteristics including age, sex, and comorbidities, which were selected using a stepwise algorithm. After controlling for selected patient characteristics, the HGLMs were applied to further evaluate the effects of hospital characteristics on hospital-level FUST and FUCAG use. The odds ratios (ORs) and corresponding 95% confidence intervals (CIs) were reported as summary statistics. The contribution of the institutional effect to hospital-level variation in FUST and FUCAGs were investigated by comparing the receiver operating characteristic (ROC) curves of the logistic regression models adjusted for only the patient case mix with the ROC curves of the HGLM models which also took into account institutional effects. The intraclass correlation coefficients were calculated. 

To evaluate the association between hospital factors and clinical outcomes, frailty models were used. After the patient characteristics which showed a significant association with clinical outcomes in Cox’s proportional hazard models were adjusted for, the frailty models were further applied to address the effects of each hospital characteristics on clinical outcomes. The hazard ratios (HRs) and corresponding 95% CIs were reported as summary statistics. 

We used SAS software version 9.4 to perform statistical analysis (SAS Institute, Inc, Cary, North Carolina), and the figures were created with R (version 3.5.1)^4^. A two-tailed *p*-value of < 0.05 was considered statistically significant.
